# A Draft Genome of the Honey Bee Trypanosomatid Parasite *Crithidia mellificae*

**DOI:** 10.1371/journal.pone.0095057

**Published:** 2014-04-17

**Authors:** Charles Runckel, Joseph DeRisi, Michelle L. Flenniken

**Affiliations:** 1 Howard Hughes Medical Institute, Chevy Chase, Maryland, United States of America; 2 Department of Biochemistry and Biophysics, University of California San Francisco, San Francisco, California, United States of America; 3 Department of Plant Sciences and Plant Pathology, Montana State University, Bozeman, Montana, United States of America; Federal University of São Paulo, Brazil

## Abstract

Since 2006, honey bee colonies in North America and Europe have experienced increased annual mortality. These losses correlate with increased pathogen incidence and abundance, though no single etiologic agent has been identified. *Crithidia mellificae* is a unicellular eukaryotic honey bee parasite that has been associated with colony losses in the USA and Belgium. *C. mellificae* is a member of the family Trypanosomatidae, which primarily includes other insect-infecting species (*e.g*., the bumble bee pathogen *Crithidia bombi*), as well as species that infect both invertebrate and vertebrate hosts including human pathogens (*e.g.,Trypanosoma cruzi*, *T. brucei*, and *Leishmania spp.*). To better characterize *C. mellificae*, we sequenced the genome and transcriptome of strain SF, which was isolated and cultured in 2010. The 32 megabase draft genome, presented herein, shares a high degree of conservation with the related species *Leishmania major.* We estimate that *C. mellificae* encodes over 8,300 genes, the majority of which are orthologs of genes encoded by *L. major* and other *Leishmania* or *Trypanosoma* species. Genes unique to *C. mellificae,* including those of possible bacterial origin, were annotated based on function and include genes putatively involved in carbohydrate metabolism. This draft genome will facilitate additional investigations of the impact of *C. mellificae* infection on honey bee health and provide insight into the evolution of this unique family.

## Introduction

The western honey bee (*Apis mellifera*) is an important pollinator of numerous economically important agricultural crops (*e.g.,* almonds, apples, melons) as well as plant species that increase the biodiversity of both agricultural and non-agricultural landscapes. Increased annual losses of commercially managed honey bee colonies have been associated with higher pathogen (viruses, bacteria, fungi, mites, trypanosomatids) incidence and abundance [1]–[3].

*Crithidia mellificae* is a trypanosomatid parasite of *Apis mellifera* that was first described in Australian bees in 1967 [4]. However, there have been very few studies characterizing this parasite or examining its effect on honey bee health in the four decades that followed its discovery. In 2009–2010 we prospectively monitored honey bee pathogens associated with 20 colonies within the context of a large-scale (>72,000 colonies) commercial beekeeping operation in the USA [5]. We determined that all 20 monitored colonies were infected with *C. mellificae* at some point during the year (April 2009 – January 2010), an average of one-third of the colonies were *Crithidia* positive at each time-point, and the relative abundance of *C. mellificae* peaked in January [5]. Further, we determined that *Crithidia mellificae* infections were strongly associated with *Nosema ceranae* and bacterial (*Spiroplasma spp.)* infections in our sample cohort [5]. This and other recent studies have resulted in a renewed interest in this pathogen. It is now appreciated that *C. mellificae* likely infects *Apis mellifera* throughout the globe. *C. mellificae* was discovered in Australia [4] and has subsequently been detected in *Apis mellifera* samples from the USA [1], [2], [5], [6], Belgium [7], China [8], Japan [9], and Switzerland [10]. In addition, *C. mellificae* infection of *Apis ceranae ceranae* was reported in China [8], although not observed in a Japanese study of *Apis ceranae japonica* [9]. Therefore additional studies are required to determine the prevalence of *C. mellificae* infections of the eastern honey bee (*Apis ceranae*).

Recent studies have correlated the presence of *C. mellificae* with colony losses in the USA and Belgium [6], [7]. Specifically, reanalysis of pooled Colony Collapse Disorder (CCD)-affected and non-CCD affected samples (from a 124 sample cohort obtained in 2006-2007) using high throughput sequencing (RNA-Seq) determined that *C. mellificae* was 6.15-fold more abundant in CCD-affected colonies [6]. Colony level analysis of the same sample cohort documented high *C. mellificae* prevalence (82.3%, n = 124), but did not correlate colony level incidence with CCD [6]. In contrast, reanalysis of a Belgium study that also had a high overall *C. mellificae* prevalence (70.5%, n = 363) found a correlation between *C. mellificae* incidence in July and over-winter colony loss (*i.e., C. mellificae* incidence of 71.3% in surviving colonies versus 81.3% in collapsed colonies) [7]. Furthermore, this study confirmed the association of *C. mellificae* infection with *N. ceranae* observed in a prospective study of colonies in the USA [5] and determined that *C. mellificae* and *N. ceranae* co-infection had a negative, synergistic impact on colony longevity [7].

The effect of C. *mellificae* on individual bees is an underexplored area of research. We expect that the draft genome presented herein will benefit future studies aimed at understanding host-pathogen interactions at the molecular level. To date, only one study has examined the honey bee host immune response to *C. mellificae* at the transcriptional level [11]. Schwarz *et al.,* 2013, exposed bees housed in a laboratory setting to *C. mellificae* in the presence and absence of *N. ceranae* and identified similarities and differences in the transcriptional profile of a panel of immune response genes [11]. Genes induced by *C. mellificae* (type strain ATCC 30254) infection included *DSCAM*, *nimrod C1*, *Imd*, *MyD88*, *abaecin*, *defensin-1*, and *defensin-2*, all of which were also up-regulated by *N. ceranae* infection [11]. Interestingly, the transcriptional profile of mixed infections differed from those observed in response to single infections [11].

Research to date suggests that *C. mellificae* infection affects honey bee health at both the colony and individual bee level. A key component to better understanding host-pathogen interactions is knowledge of the genomic sequence. The honey bee genome was sequenced in 2006 [12]. Likewise the genomes of numerous honey bee infecting viruses are known including: acute bee paralysis virus (ABPV) [13], black queen cell virus (BQCV) [14], Israeli acute bee paralysis virus (IAPV) [15], Kashmir bee virus (KBV) [16], deformed wing virus (DWV) [17], Kakugo virus (KV) [18], sacbrood virus (SBV) [19], chronic bee paralysis virus (CBPV) [20], and the Lake Sinai viruses (1–4) [5]–[7]. Additional sequenced genomes include two microsporidial pathogens *Nosema apis* [21] and *Nosema ceranae* [22], the more prevalent of the two species, and the parasitic mite *Varroa destructor* [23].

Here we present the draft genome sequence of the honey bee infecting trypanosomatid *Crithidia mellificae*, strain SF (BioProject: PRJNA78249; Accession: AHIJ00000000). Trypanosomatids are primitive unicellular eukaryotes with an unusual mitochondrial structure, the kinetoplast [24], [25]. This single large organelle contains multiple copies of the primary mitochondrial genome and thousands of copies of auxiliary genes on short, circular molecules [26]. The arrangement, replication, and ligation of this kinetoplast genome is thus far unique to this deeply rooted clade, the Kinetoplastida. In addition to this unusual organelle, at least six trypanosomatid species are known to harbor betaproteobacterial endosymbionts, some of which can be cured in culture by the presence of antibiotics [27], [28]. Consistent with their role as endosymbionts, trypanosomatid descendants rendered endosymbiont-free exhibited altered nutritional requirements [27], [29], [30].

Members of the family Trypanosomatidae infect a wide array of insect hosts, with some having a second, vertebrate, host during their lifecycle. Insect-infecting trypanosomatids include *Crithidia bombi*, which infects bumble bees (*Bombus sp*.) [31], [32], and *Crithidia fasciculata,* which infects mosquitoes [25], [33], [34]. Infections of insect hosts with these trypanosomatids cause a range of detrimental effects [24]. For example, *C. bombi* infections of *Bombus spp.* have been linked to reduced individual and colony fitness, especially under stressful conditions [31], [35], [36]. However the relationship between *C. bombi* and *Bombus terrestris* is complex, whereby specific strains of the parasite are more virulent in specific bee lineages (colonies) and infection outcome is dependent on the host microbiome [31], [32], [37]–[40].

Trypanosomatids with both insect and vertebrate hosts are of two phylogenetic lineages; the *Trypanosoma* cause human diseases such as African Sleeping Sickness (*Trypanosoma brucei*) and Chagas Disease (*Trypanosoma cruzi*); and *Leishmania spp.* cause leishmaniasis. The genomes of these human pathogens have been sequenced and well characterized [41]–[43]. Recent sequencing and analysis of trypanosomatid parasites singularly using insects as hosts contribute to our current understanding of trypanosomatid evolution and host-parasite interactions; these analyses will be enhanced by ongoing sequencing and annotation efforts [24], [27]–[29]. Phylogenic analyses of the Trypanosomatidae place the vertebrate pathogens of genus *Leishmania* intermediary to the insect trypanosomatid clade and the Trypanosoma clade, which includes *T. brucei* and *T. cruzi* [8], [24], [27], [44]–[48]. Therefore we performed the majority of our analyses of the draft genome of the honeybee trypanosmatid parasite *C. mellificae* as a comparison with the well-annotated genome of *L. major* [42].

*Crithidia mellificae* is a trypanosomatid parasite of honey bees that is currently under-characterized given its potential role and recent association with colony losses in the USA and Belgium [6], [7]. The genome of this gut parasite provides an important foundation for further molecular, evolutionary, and epidemiological characterization of this potential threat. The 32 megabase draft genome sequence presented herein shares a high degree of conservation with the related species *Leishmania major.* We estimate that *C. mellificae* encodes over 8,300 genes. The majority of *C. mellificae* genes are orthologous to genes encoded by *L. major* (84%) and other *Leishmania* or *Trypanosoma* species (8.1%). In addition, we illustrate conservation of genomic features such as directional gene arrays and a lack of intron-spliced genes. Genes unique to *C. mellificae,* including those of possible bacterial origin, were annotated based on function and include genes putatively involved in carbohydrate metabolism. Characterization of genes gained and lost in *C. mellificae* compared to related lineages will lead to a better understanding of the evolutionary pressures operating at the host-pathogen interface. Furthermore, this species and other *Crithidia spp.* have an impact on social bees that are key to both agricultural pollination and pollination in natural settings. The *Crithidia* genome sequence described herein will assist efforts to better understand host-parasite interactions and may lead to strategies that mitigate its impacts on pollinator health.

## Results

### *Crithidia mellificae* genome sequencing and comparison with *Leishmania major*

**Figure 1:**
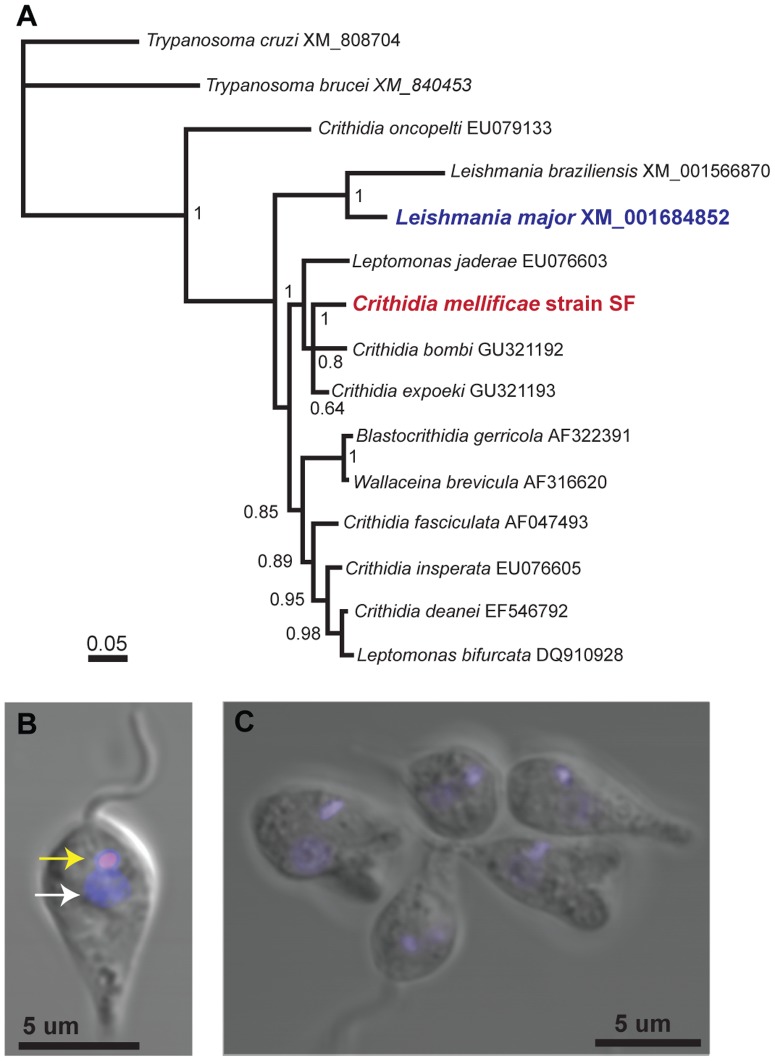
*Crithidia mellificae*, a trypanosomatid parasite of honey bees. (A) Majority consensus tree of select members of the Trypanosomatidae derived from Bayesian analysis [55], [56] (*i.e.,* MrBayes v3.1.2 [57]) of a *glyceraldehyde 3-phosphate dehydrogenase* (*GAPDH*) nucleotide alignment (799 nt). *T. cruzi* was selected as the outgroup based on results from previous phylogenetic analyses [15], [39], [50]–[52]. Numbers on branches are Bayesian posterior probabilities (0–1); scale bar corresponds to the proportion of nucleotide change. The genus and species names are consistent with the GenBank accession numbers in the figure; we note that *Crithidia deanei* was renamed *Angomonas deanei*. (B) Composite of light and fluorescent microscope images of *C. mellificae* illustrate the flagellum, kinetoplast (smaller, brighter DAPI stained organelle; yellow arrow) and nucleus (white arrow) of the crithidial stage and (C) additional life-stages in culture.

*Crithidia mellificae*, strain SF, was isolated from the intestines of infected bees and grown in culture (strain SF, ATCC PRA-403) [5]. Comparison of the *glyceraldehyde 3-phosphate dehydrogenase* (*GAPDH*) gene from this strain determined that the nucleotide sequence over this region was identical to the BruceSD_T17 strain (JF423199) previously described [5], and shared in 99.8% identity with additional *C. mellificae GAPDH* sequences deposited in the NCBI database (AB716357, AB745489) (Figure S1). To place *Crithidia mellificae* in context, we utilized the nuclear encoded *GAPDH* gene nucleotide (nt) sequence, as opposed to the kinetoplast-encoded *cytochrome b* gene (*Cyt b*) reported in Morimoto, *et al.,* 2013 [9], for phylogenetic analysis of select members of the Trypanosomatidae family (Figure 1A). Characteristic features of trypanosomatids include the presence of a single flagellum and a kinetoplast, which is a structure containing a DNA network consisting of multiple copies of the mitochondrial genome. These structures are easily visualized in composites of light and fluorescent microscope images of the epimastigote or crithidial stage, which is morphologically distinguished as having an anterior flagellum and a kinetoplast located between the anterior end and the nucleus (Figure 1B) [24], [49]. The epimastigote form was predominant in culture, however multiple life stages were visualized by microscopy (Figure 1C).

**Figure 2:**
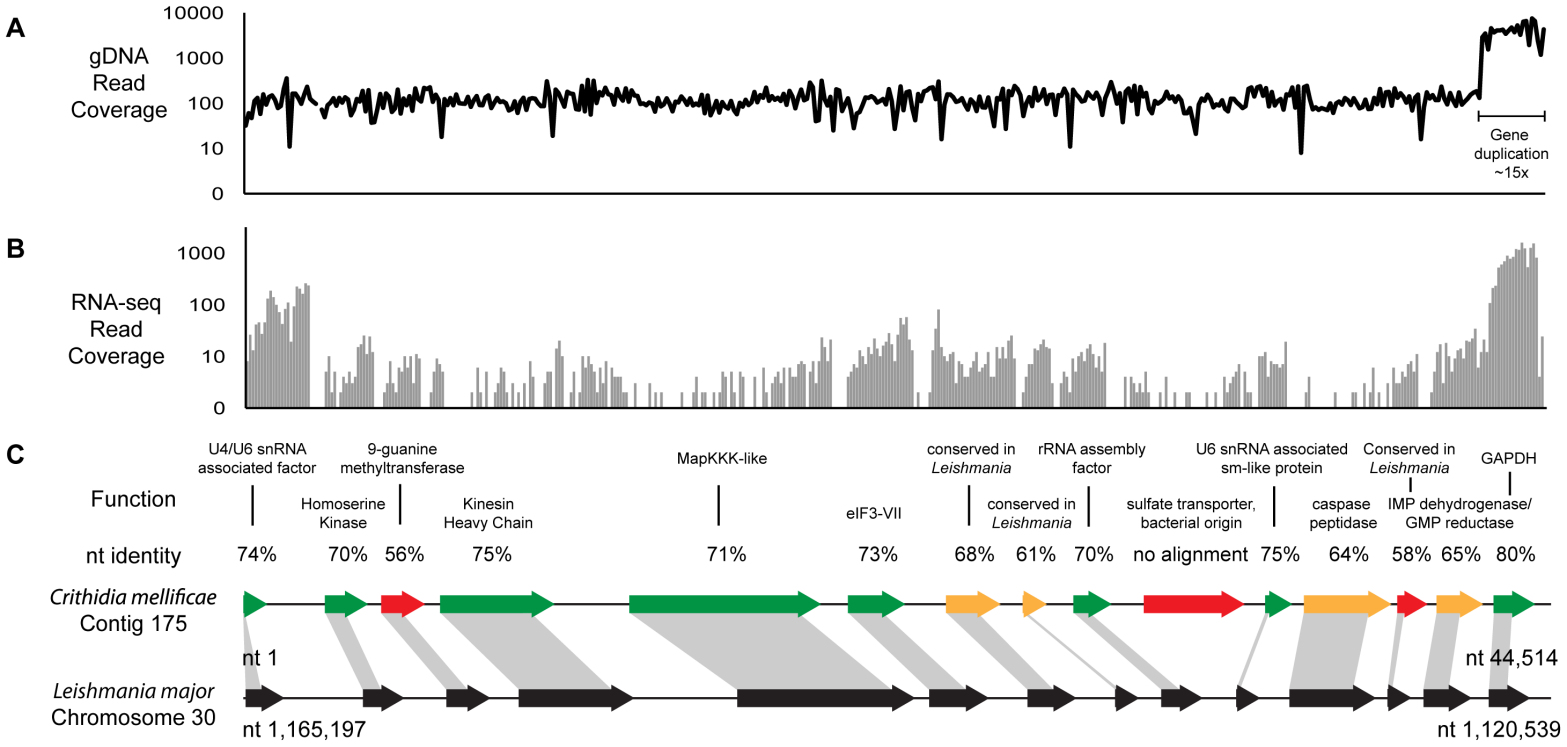
Assembly and annotation of *C. mellificae* contig 175. (A) Read coverage of the gDNA library used for assembly. A gene duplication of the *GAPDH* gene is highlighted on the right. (B) RNA-seq coverage aligned to the contig. (C) Genes predicted by the Maker pipeline in *C. mellificae* with assigned putative functions. The homologous and syntenic region of *L. major* is shown below, with nucleotide identity of the *C. mellificae* genes to *L. major* color-coded by nt identify (green ≥70%, yellow ≥60%, red <60%). A putative bacterial xenolog of a sulfate transporter, sulfate permease JQ247792, is noted (sixth from the right).

Genome and transcriptome sequencing libraries were prepared from DNA and RNA that was isolated from cultured *Crithidia mellificae*, strain SF. Sequencing of the genomic DNA (gDNA) library was performed on an Illumina Genome Analyzer IIx with a V3 paired-end cluster generation kit and V5 sequencing reagent and complementary DNA (cDNA) library was sequenced on an Illumina HiSeq 2 with V2 and V3 paired-end chemistry. Prior to assembly, the sequencing data were filtered to remove paired reads that contained more than five ambiguous bases in either read. The *Crithidia mellificae* genome was assembled using only short, paired-end Illumina reads (65 nt) at ∼100× coverage (Table 1, Figure 2A) using the ABySS [50], PRICE [51], and Geneious [52] assemblers. Operationally, we used ABySS to produce initial short contigs, and then PRICE, a local assembler, to extend and join these contigs in sub-pools, and finally Geneious as a sequence workbench for final assembly and manipulation. This combination increased the N50 contig size by 12-fold compared to the original output of ABySS alone. In total the draft genome is 32 megabases (mb) (GenBank AHIJ01000000) in length with an N50 metric of over 32 kb (Figure S3). A similar pipeline was used to assemble the transcriptome. After genome and transcriptome assembly, genes were predicted by the Maker package [53] based on *ab initio* predictions, assembled EST evidence, or protein alignment to all trypanosomatid proteins in GenBank (BioProject: PRJNA78249; Accession: AHIJ00000000) [54]. An example contig with corresponding gDNA, RNA coverage, and gene predictions is shown in Figure 2.

**Table 1:** *Crithidia mellificae* genome assembly statistics.

Crithidia mellificae	Assembly Statistics
**Input Reads**	29,004,448 clusters
**Read Length**	65 nt paired
**# Contigs**	2,801
**Assembly Size**	32,812,984 nt
**N50**	32,366 nt
**% Reads Aligned**	91%
**# Genes predicted**	9,979
**Total coding sequence**	18,265,100 nt
**Gene median length**	1,470 nt
**Total GC Content**	53.5%
**Coding GC Content**	62.4%
**Intergenic GC**	42.3%

The Trypanosomatidae family was first described using a morphotype-based taxonomic system (reviewed in Maslov *et al,.* 2013 [24]). More recently sequence based phylogenetic analysis of SSU rRNA and protein phylogenies [46], [47] indicate that *T. brucei* and *T. cruzi* are part of the Trypanosoma clade of Trypanosomatida, with the Leishmania branch intermediary to the insect trypanosomatid clade. Likewise maximum likelihood (ML) analysis based on the combined SSU rRNA and *gGAPDH* gene sequences of symbiont harboring trypanosomatids placed the *Leishmania major* clade more proximal to the *Crithidia fasciculata* containing clade, as compared to the *Trypanosoma cruzi* containing clade [27]. The phylogenetic relatedness of *Crithidia spp. and Leishmania spp.* also holds in protein-based phylogenic analyses using three-concatenated protein sequences (pteridine reductase, γ-glutamylcysteine synthetase, and adenine phosphoribosyl transferase) [48]. Likewise, phylogenetic analyses of the kinetoplast-encoded *cytochrome b* (*Cyt b*) gene from *C. mellificae* isolates in Japan and China using maximum likelihood method under the Tamura 3-parameters with a discrete gamma distribution model also places *Leishmania spp.* intermediary to *Trypanosoma spp.* and *Crithidia fasciculata* [8], [45]. To place *C. mellificae* in phylogenetic context we implemented Bayesian inference [55], [56] utilizing MrBayes v3.1.2 [57] to infer phylogenetic relatedness using a *GAPDH* alignment and selecting *T. cruzi* as the outgroup based on results of previous phylogenetic analyses [8], [9], [24], [27], [29], [46]–[48] (Figure S2). The Bayesian majority-rule consensus indicated that the vertebrate-infecting genus *Leishmania* is closely related to several insect-infecting trypanosomatids including *C. mellificae*; and that other vertebrate-infecting trypanosomatids, such as the genus *Trypanosoma*, are basal to them (Figure 1A and more thoroughly treated in Hughes and Piontkivska, 2003 [44], Teixeira *et al.,* 2011 [27], and others [29], [46]–[48]).

*Leishmania major* is the most closely related trypanosomatid that has been completely sequenced and annotated, thus it was used for the comparative analysis of the *C. mellificae* genome described herein. A total of 9,971 coding sequences are predicted in this draft genome of *C. mellificae*, including truncated genes at the edges of contigs that will exaggerate the total gene count by double counting split genes. Specifically, 17% of the genes that were identified using blastx [58] (a translated nucleotide query used to probe the NCBI non-redundant protein database) to have a specific identify and function were non-overlapping duplicate search matches located at the truncated ends of contigs and thus predicted as individual coding sequences. These are presumably single incompletely assembled genes, suggesting that the actual gene count is ∼8,300 coding sequences, in line with the 8,265 genes identified in the complete *Leishmania major* genome [42]. The decision to include truncated coding sequences at contig ends falsely increased the total gene count, but also increased the proportion of conserved trypanosomatid genes that can be accounted for and thus favored detection of gene gains or losses at the cost of overestimated gene numbers. Despite the inclusion of truncated genes, the total length of coding sequences (18.3 mb vs. 15.7 mb) and the median coding sequence length (1,470 nt vs. 1,428 nt) are comparable between this assembly and the *L. major* genome.

Previously sequenced *Leishmania* genomes are notable for their conservation of synteny and stretches of shared directionality over dozens of adjacent genes (Figure 2C); presumably, both traits are indications of their reliance on gene arrays for transcriptional control [8], [42], [59]. The conservation of synteny is substantial; there are only four synteny breaks observed in the ten largest *C. mellificae* contigs (containing 488 genes and 1.5 megabases of sequence) compared to the *L. major* genome. Shared directionality between adjacent genes was not used in the assembly process and is thus an unbiased statistic; in those ten contigs adjacent gene pairs exhibit a 98.5% chance of sharing the same coding strand.

### Unique *C. mellificae* genes relative to trypanosomatids

**Figure 3:**
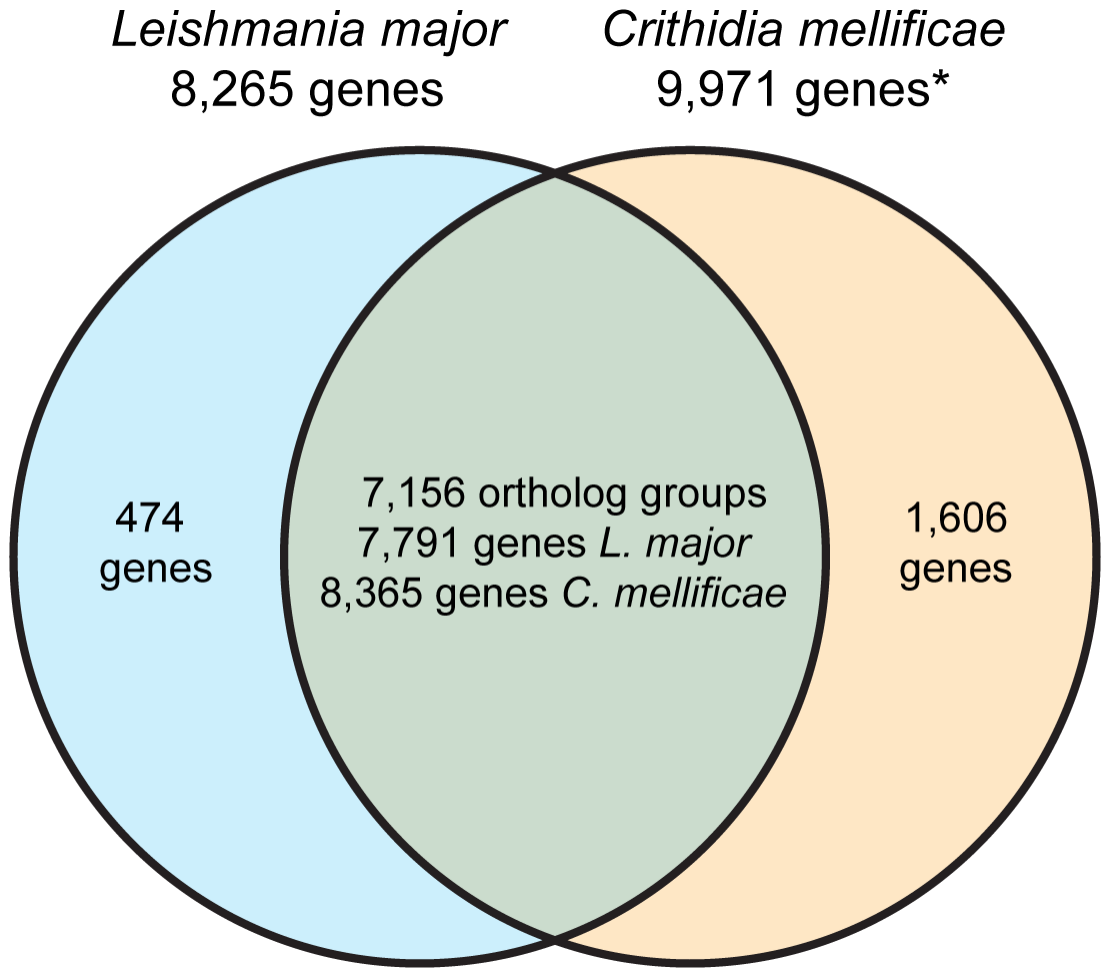
The gene catalogues of *Leishmania major* and *Crithidia mellificae* are compared after ortholog analysis by INPARANOID [60]. *Truncated genes at contig ends were included in this analysis for a total of 9,971 ORFs. Approximately 17% of these ORFs are incomplete ends of the same presumed gene, resulting in ∼8,300 actual genes (see Results).

Orthologs of *C. mellificae* genes were identified in *L. major* by the INPARANOID algorithm [60], based on reciprocal BLAST alignments and ortholog clustering. This analysis indicates that 474 of the 8,265 (5.7%) predicted genes in *Leishmania major* lack an ortholog in *C. mellificae.* The majority of *Crithidia*-absent genes lack an annotated function, with the remainder being a diverse set that lacks a significant enrichment for any particular function or process. In contrast, 8,365 of the 9,971 *C. mellificae* genes (84%) possess orthologs in *Leishmania major*, and an additional 805 (8.1%) of the predicted *C. mellificae* genes that lack an ortholog in *L. major* matched proteins annotated as *Leishmania* or *Trypanosoma* in the NCBI non-redundant database (nr) (Figure 3). Of the remaining predicted proteins, several align to another organism in the nr database (Table 2) while the majority did not match any annotated protein. Of those that matched a non-trypanosomatid, the top tblastx alignments for three genes are to eukaryotic proteins, and an additional 13 genes best align to bacterial proteins (Table 2). The majority of *C. mellificae* genes of putative bacterial origin are flanked by genes otherwise syntenic in *L. major* and share directionality with adjacent genes (for example Figure 2C “sulfate transporter”), suggesting that putative bacterial genes are a part of transcriptionally regulated gene arrays rather than erroneous assembly artifacts of environmental contaminants. Furthermore, re-mapping of paired-end reads to the contigs containing genes of putative bacterial origin did not reveal discrepancies in assembly or alterations in coverage level (Figure 2), as would be expected for a contaminant incorporated by mis-assembly. In addition, the GC content of putative bacterial genes (60.9% GC) is similar to the coding region of the *Crithidia mellificae* genome (62.4% GC). Together, these results support the notion that the genes we identified within our assemblies that were of putative bacterial origin are not the product of contamination and mis-assembly.

**Table 2:** Predicted genes unique to *Crithidia mellificae.*

EC Number	Gene *C. mellificae*	Accession	Function	Putative Origin	tblastx*
3.2.1.21	beta-glucosidase B	JQ247767	carbohydrate metabolism	bacteria	*C. mellificae* only
-	ABC-type sugar transporter	JQ247763	carbohydrate metabolism	bacteria	Trypanosomatidae chromosomes
1.14.11.18	pytanoyl-CoA dioxygenase	JQ247790	carbohydrate metabolism	bacteria	*C. mellificae* only
1.1.1.289	NADPH-dependent l-sorbose reductase	JQ247784	carbohydrate metabolism	bacteria	Trypanosomatidae chromosomes
1.1.1.27	l-lactate dehydrogenase	JQ247781	carbohydrate metabolism	bacteria	Trypanosomatidae chromosomes
3.1.1.17	glucolactonase	JQ247775	carbohydrate metabolism	bacteria	*C. mellificae* only
3.5.3.1	arginase	JQ247765	urea/polyamine processing	bacteria	*C. mellificae* only
-	sulfate permease	JQ247792		bacteria	*C. mellificae* only
1.5.3.1	sarcosine oxidase	JQ247791	glycine, serine, threonine metabolism	bacteria	Trypanosomatidae chromosomes
2.3.1.118	n-hydroxyarylamine o-acetyltransferase	JQ247783		bacteria	Trypanosomatidae chromosomes
-	flavohemoprotein	JQ247773	oxidoreductase	bacteria	*C. mellificae* only
-	PfkB domain-containing protein	JQ247787	carbohydrate metabolism; kinase	bacteria	Trypanosomatidae chromosomes
-	ADP-ribosylation crystalline J1	JQ247764		bacteria	Trypanosomatidae chromosomes
-	intracellular chloride channel-like	JQ247780		eukaryote	Trypanosomatidae chromosomes
-	inositolphosphoryl-ceramide-b/fatty acid hydrolase/FAD-dependent oxidoreductase	JQ247778	fatty acid metabolism	eukaryote	Trypanosomatidae chromosomes
3.4.23.24	cathepsin-like protein/aspartyl protease	JQ247769	protein degradation	eukaryote	Trypanosomatidae chromosomes

*tblastx of each predicted gene was performed using all Trypanosomatidae sequences in the nr database, a tblastx threshold of an E-value ≤10^−6^ was selected and alignments that scored within this threshold and had an associated accession number and annotation are reported above. Significant alignments are reported as follows, “*C. mellificae* only” indicates that no other annotated sequence aligned with this gene, “Trypanosomatidae chromosome” indicates additional unannotated chromosomal sequences from trypanosomes, often very large data files, have a region within them that aligns with the annotated sequence from *C. mellificae*.

Sugar metabolism in trypanosomatids is carried out in catalase-deficient peroxisomes called glycosomes, which are specialized metabolic organelles for glycolysis and pentose processing. Analysis of the *Crithidia mellificae* genes with no significant protein ortholog included several involved in carbohydrate metabolism (Table 2). We identified a *beta-glucosidase B* (JQ247767) gene unique to *Crithidia mellificae* and an *ABC-type sugar transporter* (JQ247763) gene of putative bacterial origin that aligns with unannotated sequence of other Trypanosomatidae family members (Table 2). Further, Opperdoes and Michels (2007) previously identified 42 *L. major* genes of suspected bacterial origin and 14 genes of plant or cyanobacterial origin, most of which are involved in sugar metabolism; all but one have orthologs in the *C. mellificae* genome [61]. The core genes involved in glycosomal glycolysis and succinate production are all conserved between *L. major* and *C. mellificae*.

**Figure 4:**
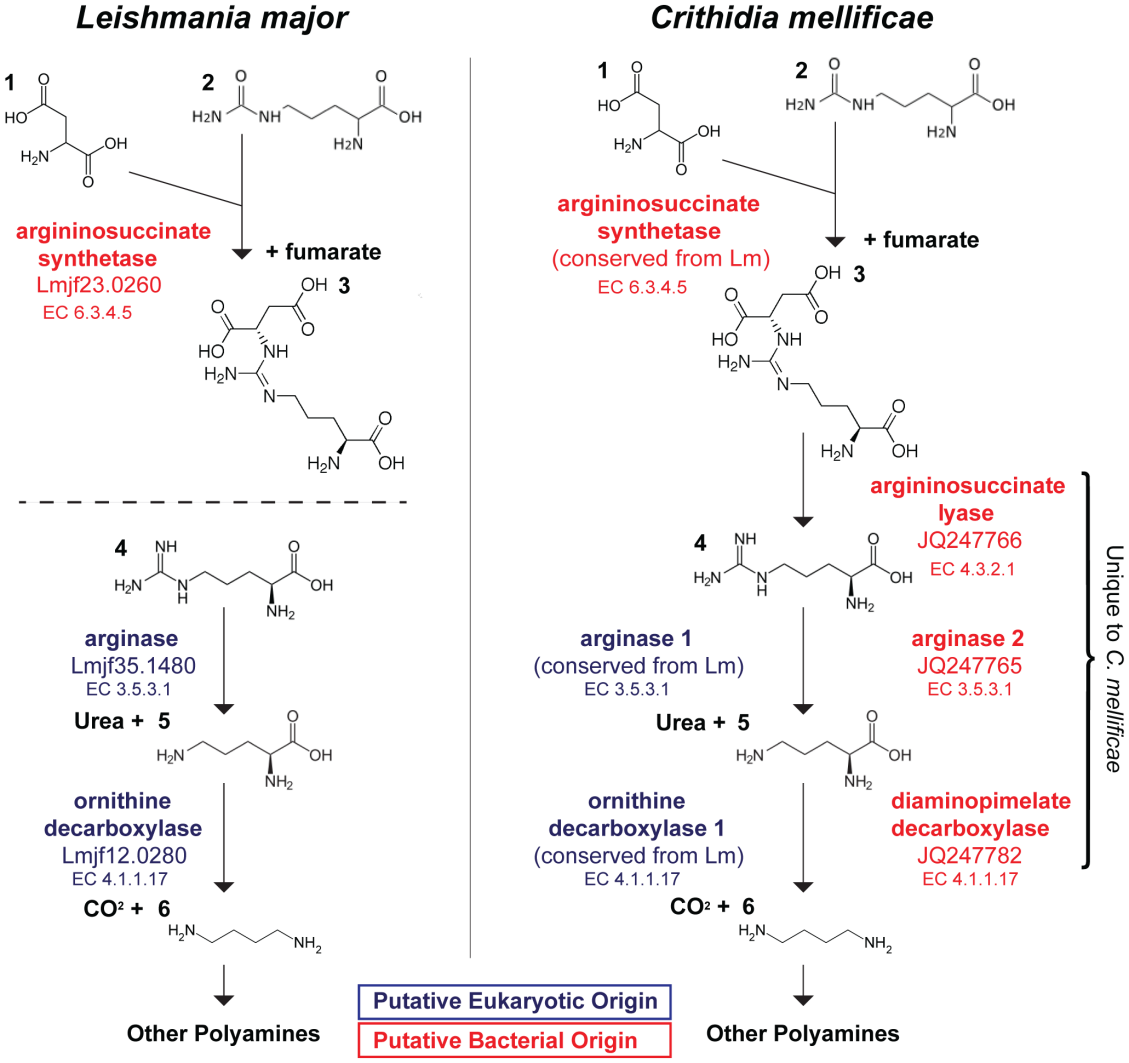
Urea and polyamine synthesis in trypanosomatids. The putative origin of genes involved in urea and polyamine synthesis is indicated by color; eukaryotic (blue) or bacterial (red) origin. For *C. mellificae*, genes conserved from *L. major* are displayed on the left column and unique genes on the right; the compounds are numbered as follows: 1. aspartic acid, 2. citrulline, 3. argininosuccinate, 4. arginine, 5. ornithine, 6. putrescine.

Alves *et al*., 2013 analyzed the evolutionary origin of genes involved in amino acid synthesis of several trypanosomatids including *Crithidia acanothocephali* (TCC037E) [29]. Our results are consistent with this study, we identified several *C. mellificae* genes involved in polyamine synthesis of putative bacterial origin and the Trypanosomatidae orthologs of these genes using tblastx (Figure 4) [29], [61]. Specifically, diaminopimelate decarboxylase (JQ247782; orthologs include: *C. acanthocephali* KC545154, *S. oncopelti* KC545099, *S. galati* KC545214, *S. culicis* KC476502, *H. muscarum* KC503401.1, *A. deanei* KC503345.1, *A. desouzai* KC584076.1), diaminopimelate epimerase (JQ247771; *H. muscarum* KC503402, *L. infantum* FR796460, *L. donovani* FR799615, as well as alignments with *L. major*, *L. braziliensis*, and *L. mexicana* chromosomes), argininosuccinate lyase (JQ247766; *C. acanthocephali* KC545122, *A. desouza*i KC545122, *S. galati* KC545178, *A. deanei* KC503302, *S. culicis* KC140155, *S. oncopelti* KC545050, *H. muscarum* KC503365), whereas only unannotated regions of Trypanosomatidae chromosomal sequences aligned to the *C. mellificae* arginase (JQ247765) identified herein. As more extensively discussed by Alves *et al*., 2013 this pathway likely represents a bacterially-derived alternative pathway for the production of putrescine from argininosuccinate, which is in turn a substrate for growth-limiting polyamine production [29], that is present in *C. mellificae* and absent in *L. major*.

### Splicing

Cis-splicing of introns is extremely rare in previously studied trypanosomatids [62]. To investigate the potential for cis-splicing in *C. mellificae* we used HMMSplicer [63] and long, paired-end transcriptome sequencing (2×100 nt reads with a 300–350 insert size) to identify potential splicing events in *C. mellificae*. Reads were mapped to the assembled genome and possible splicing events scored. No junction scored higher than 1000, despite high read coverage, indicating low confidence. Twelve proposed events were randomly selected from the top scoring bin (900–1000) and investigated by RT-PCR; however, none could be confirmed. In addition, we determined that the *C. mellificae* orthologs of the four intron-containing genes in *L. major* (LmjF.29.2600 poly(A) polymerase, LmjF07.3400 ATP-dependent DEAD/H RNA helicase, LmjF29.2000 hypothetical protein conserved, and a LmjF32.0850 putative RNA-binding protein [42]) are encoded by contiguous *C. mellificae* sequence by blasting the coding sequences against the genome sequence (Figure S4). Our results do not completely rule out the existence of cis-spliced genes in this organism, but indicate they are likely to be rare.

## Discussion

*Crithidia mellificae* is a trypanosomatid parasite of honey bees that is prevalent in samples obtained throughout the globe. Recent correlations between *C. mellificae* abundance and CCD [6], and *C. mellificae* incidence and over-winter colony loss [7] underscore the importance of better characterizing this parasite and understanding its role in honey bee health. In this work, we describe the first draft genome of *Crithidia mellificae.* We employed a hybrid, guided assembly strategy based on an initial assembly by whole-dataset de Bruijn graph assemblers (*i.e.,* ABySS) [50]) joining these contigs by inference based on gene-level synteny with related organisms, and contig extension with a targeted assembler, PRICE [51].

The *Crithidia mellificae* genome shares remarkable similarity in structure and gene content with relatives of the genus *Leishmania*, which infect both invertebrate and vertebrate hosts. High order structures such as gene arrays with large blocks of shared directionality are conserved despite large evolutionary divergence and a host shift from the ability to infect only insects to both insects and vertebrates. We did not identify any evidence of cis-splicing of introns by *ab initio*, transcriptome, *L. major* guided searches, or by RT-PCR of candidates. These results do not rule out the possibility of cis-spliced genes in *C. mellificae*, but indicate that splicing is rare.

The majority of the genes that are absent in the *Crithidia* lineage, as compared to *Leishmania*, are poorly classified and identifiable genes were not significantly enriched for any function or process. The majority of genes identified in *C. mellificae*, but not found in *L. major* have putative bacterial origin. These genes share directionality with their neighbors, have uniform sequence coverage, and similar GC content, strongly supporting the notion that these genes are indeed resident in the genome rather than being spurious products of contamination.

Alves *et al.,* 2013 performed an evolutionary analysis of genes involved in amino acid biosynthesis and metabolic pathways in trypanosomatids [29]. Our results our consistent with this study, we identified several *C. mellificae* genes involved in polyamine synthesis that are of putative bacterial origin [29], [64], as well as their Trypanosomatidae orthologs. In this work we identified *C. mellificae* arginase (JQ247765), tblastx analysis of this gene query to the nr database resulted in significant alignments with bacterial encoded arginases, whereas the same query limited to Trypanosomatidae sequence data resulted in no significant alignments (E-value ≤10^−6^), only weak alignments to cloned or chromosomal sequences deposited in the NCBI database (*i.e., S. galati*, E-value  = 0.010 and *Leishmania spp.*, E-value  = 0.003). Sequencing of additional trypanosomatids and continued annotation of existing genome sequences, particularly other *Crithidia* species, will expand our understanding of this group of organisms and the interesting role of horizontal gene transfer in the evolution of the metabolic processes required to adapt to specific niches [29], [61].

The majority of genes identified herein as unique to *Crithidia mellifcae*, and of putative bacterial origin, likely function in carbohydrate metabolism (Table 2). This is particularly interesting as previous work also determined that *Leishmania* genes of putative bacterial origin, most of which were conserved in *Trypanosoma* genomes, were involved in sugar intake and metabolism [61]. It is thought that these genes were acquired via horizontal transfer from an endosymbiont, early in the trypanosomatid lineage; trypanosomatids first colonizing the insect digestive tract, a sugar-rich environment, would have required novel metabolic genes. All but one of those genes are conserved in *C. mellificae*, along with additional bacterial sugar processing genes (Table 2). In addition, our analysis of genes that are unique to *Crithidia mellificae* revealed three genes of putative eukaryotic origin including an Intracellular chloride channel-like (JQ247780) protein with a top tblastx alignment to protein encoded by the single-celled green algae *Chlorella*, Inositolphosphoryl-ceramide-b with a top tblastx alignment to the sponge protein ortholog, and a Cathepsin-like protein (JQ247769) with a top tblastx alignment to a Cathepsin protein in nematodes (Table 2). Clearly annotation of all Trypanosomatid genomes will be furthered by additional analysis of existing genomes and the completion of several genome projects currently underway [8], [24], [27], [28], [44]–[48].

The *Crithidia mellificae* draft genome described herein will further our understanding of trypanosomatids and the evolutionary pressures operating at the host-pathogen interface. It will also facilitate further investigation of the effects of *C. mellificae* on honey bee health. Future studies aimed at understanding the role of parasites in the context of other common pathogens and environmental stress factors (*e.g.,* chemical and nutritional) at both the individual bee and colony level are critical toward understanding recent honey bee colony losses. Honey bees are important pollinators of plants in both agricultural and non-agricultural landscapes, thus strategies that mitigate negative impacts on pollinator health are essential for global food production and the maintenance of biodiversity.

## Materials and Methods

### *Crithidia mellificae* culture and nucleic acid preparation

Modern *Crithidia mellificae* (strain SF, ATCC PRA-403) isolates were collected and cultured as previously described [5]. In brief, honey bees from a colony previously determined to be *Crithidia* positive by PCR were obtained with permission from privately owned, managed colonies in San Francisco (SF), CA. No additional permissions were required since the managed honey bee colony was privately owned and *Apis mellifera* is not an endangered or protected species. Bees were chilled at 4°C and washed in 70% EtOH prior to decapitation and dissection under sterile conditions. Minced intestine was cultured in BHT medium composed of Brain Heart Infusion (BHI) 28.8 g/L (DIFCO), tryptose 4.5 g/L (DIFCO), glucose 5.0 g/L, Na_2_HPO_4_ 0.5 g/L, KCl 0.3 g/L, hemin 1.0 mg/L, fetal bovine serum (heat inactivated) 2% v/v, pH 6.5, and containing penicillin G sodium (106 units/L) and streptomycin sulfate (292 mg/L) at 27°C [65]. Free active cells were observed 24 hours post inoculation. Parasites were maintained by subculture passage every 4 days; stable liquid nitrogen stocks were archived. Light microscopy of live parasites was performed using a Leica DM6000 microscope equipped with Hamamatsu C4742-95 camera and Volocity Software (PerkinElmer). Imaging fixed parasites (4% paraformaldehyde, 20 min) facilitated visualization of DAPI (4′,6-diamidino-2-phenylindole) stained nuclear and kinetoplast DNA. Images of fixed *C. mellificae* were obtained using both the Leica DM6000 microscope and a Zeiss LSM 510-M microscope equipped with both a 636 objective numerical aperture 1.4, and a 1006 objective numerical aperture 1.4. For DNA purification, *Crithidia mellificae* (∼10^6^ trypanosomes/mL culture medium) were pelleted by centrifugation (800×g for 6 min) and washed with phosphate buffered saline (PBS) prior to DNA extraction. DNA was extracted using the DNeasy Genomic DNA Extraction Kit (Qiagen) as per the manufacturer's instructions.

### Illumina sequencing

DNA and RNA libraries were generated by transposase-mediated fragmentation and adapter ligation using the Nextera DNA Sample Prep Kit - Illumina-compatible (Epicentre). gDNA was used directly (50 ng) while total RNA digested with Turbo DNAse (Ambion) prior to reverse transcription with SuperScript III (Invitrogen) with an oligo dT primer and a second strand generated by Sequenase (USB) with a random hexamer primer (100 ng of ds-cDNA was used for the downstream reaction). Library preparation was performed as per manufacturer's instructions, except that the PCR was paused at 5 cycles instead of the recommended 8 and the product run on a 8% native acrylamide TBE gel (Invitrogen) and a band excised in the 300–350 nt range for the gDNA library (150–200 nt insert size) followed by recovery by electroelution. The cDNA library was run on the LabChip XT (Caliper) and extracted in the 450–500 nt range (300–350 insert size). After size selection, five additional cycles of PCR were performed. Sequencing of the gDNA library was performed on a single lane of an Illumina Genome Analyzer IIx with a V3 paired-end cluster generation kit and V5 sequencing reagent. The cDNA library was sequenced on an Illumina HiSeq 2 with V2 and V3 paired-end chemistry.

### Assembly

The sequencing data were filtered and paired reads removed if more than five ambiguous bases were present in either read. The reads were then assembled in a single pool by the Abyss-pe assembler (v1.27, Simpson, *et al*., 2009 [50]) and manipulated in the Geneious sequence workbench (v5, [52]), including identification and extraction of ORFs. Sequences were aligned to 100 kb segments of the *Leishmania major* genome by blastx [58] and sub-pools were extended and combined with the PRICE assembler [51]. Reads that did not align to the current assembly were identified by blastn, extracted, and the pipeline repeated. The Geneious assembler was then used to identify overlapping contigs prior to additional assembly by PRICE.

### Annotation and analysis

The Maker pipeline [53] was used to annotate the draft genome, initially using the nucleotide alignment of the transcriptome data (blastn ≤E^−10^), alignment of all proteins in GenBank annotated as *Trypanosoma* or *Leishmania* (blastx ≤E^−6^) and *ab initio* gene predictions using Augustus with the *Leishmania tarentolae* gene model. The results of the first pass were used to retrain and run the annotation pipeline again. Contigs were then manually examined and additional predicted coding sequences selected based on any three of the following four criteria: 1.) open-reading frame >300 nt, 2.) ORF overlaps a CpG island [66], 3.) transcriptome alignment or 4.) protein alignment from *Leishmania* and *Trypanosoma*. ORFs that were potentially truncated at the edge of a contig were also annotated if they matched these criteria. CDS annotations were manually removed from repeat regions. Ontology analysis of predicted genes was performed by Blast2GO [67] using Blast and InterProScan [68] and manually using HHsearch with the pfam-A database and PSI-BLAST using the nr database. In addition to the program Exonerate used in the Maker pipeline, HMMsplicer [63] was used to identify and score possible splice sites and alternately spliced transcripts. The INPARANOID algorithm of reciprocal blast searches and clustering was employed as described [60]. The annotated draft genome and transcriptome are available in the National Center for Biotechnology Information (NCBI) GenBank database (BioProject: PRJNA78249; Accession: AHIJ00000000).

### Phylogenetic analysis

The *Trypanosomatidae* phylogeny (Figure 1) was inferred using Bayesian inference [55], [56] as implemented in MrBayes v3.1.2 [57] using a *GAPDH* alignment (Figure S2) and selecting *T. cruzi* as the outgroup based on results of previous phylogenetic analyses [24], [29], [46]–[48]. The best-fitting nucleotide substitution model (GTR + I + Γ) with base frequency, substitution rates, and among-site variation variables was estimated from the data via the Akaike information criterion (AIC) [69] as implemented in ModelTest 3.7 [70]. Metropolis-coupled Markov Chain Monte Carlo (MCMC) permutation of parameters were initiated with a random tree and involved two runs each with four chains set at default temperatures [71]. Markov chains were run for 5,000,000 generations and sampled every 50,000th generation such that 200 nonautocorrelated Bayesian trees were sampled broadly from likelihood stationarity for each of the two runs after a burn-in. MrBayes was used to assess the convergence of the MCMC run and the adequacy of the burn-in length. Trees sampled from post burn-in generations were summarized in a majority rule consensus tree that included posterior probabilities as branch support estimates. The Bayesian majority-rule consensus was then visualized and partially edited using FigTree v1.4.0 (Rambaut, 2012) and Geneious [52].

### Splice junction verification

Putative splice sites were identified by HMMsplicer and grouped by output score into 100-point bins. Twelve candidates were selected from the highest bin, with scores of 900–1000. PCR primers were designed by Primer3 to span the putative splice junction and generate ∼300 nt PCR amplicons if a splicing had occurred. RT-PCR was performed with Onestep SuperScriptIII and PlatinumTaq (Invitrogen) from DNAse-treated total RNA generated for the initial transcriptome library, with 100 ng of RNA used per reaction. PCR reactions were run for 40 cycles with an annealing temperature of 58°C, and all bands in the 100–1000 nt range were cut and extracted from a 2% agarose gel and cloned by Topo-TA (Invitrogen) prior to colony PCR and sequencing on a 3310xl Genetic Analyzer (ABI).

## Supporting Information

Figure S1 Nucleotide alignment of all *C. mellificae GAPDH* sequences in NCBI (JF423199, AB716357, AB745489); these sequences are 99.8% identical.
(PDF)

Figure S2 Nucleotide alignment (799 nt) of the *glyceraldehyde 3-phosphate dehydrogenase* (*GAPDH*) gene. The alignment was performed on the Geneious software workbench, using the ClustalW aligner.
(PDF)

Figure S3 N-value assembly metric of the *C. mellificae* draft genome.
(PDF)

Figure S4 *C. mellificae* orthologs of spliced *L. major* genes.
(PDF)
