# Supplementary material for: A Draft Genome of the Honey Bee Trypanosomatid Parasite Crithidia mellificae
Source: PLoS One. 2014 Apr 17;9(4):e95057. doi: 10.1371/journal.pone.0095057 (PMC3990616; doi:10.1371/journal.pone.0095057)
Supplement: Figure S1 — Nucleotide alignment of all C. mellificae GAPDH sequences in NCBI (JF423199, AB716357, AB745489); these sequences are 99.8% identical. (PDF) [file pone.0095057.s001.pdf]

Supporting Figure S1. Nucleotide alignment of all *C. mellificae* GAPDH sequences in NCBI (JF423199, AB716357, AB745489); these sequences are 99.8% identical.

```
                                        1        10        20        30        40
1. Crithidia mellificae strain SF GAPDH ATGGCTCCCCTGAAGGTTGGCATCAACGGTTTCGGCCGCATTGGCCGC
2. Crithidia mellificae JF423199
3. Crithidia mellificae AB716357
4. Crithidia mellificae AB745489

                                         50        60        70        80        90
1. Crithidia mellificae strain SF GAPDH ATGGTGTTCCAGTCGATGTGCGAGGGCGACGTCCTCGGCAAGGAGATC
2. Crithidia mellificae JF423199                                                  GAGAUC
3. Crithidia mellificae AB716357
4. Crithidia mellificae AB745489

                                           100       110       120       130       140
1. Crithidia mellificae strain SF GAPDH GACGTGGTCGCCGTGGTGGACATGAGCACGGACGCCGAGTACTTCGCG
2. Crithidia mellificae JF423199        GACGUGGUCGCCGUGGUGGACAUGAGCACGGACGCCGAGUACUUCGCG
3. Crithidia mellificae AB716357
4. Crithidia mellificae AB745489                        TGGACATGAGCACGGACGCCGAGTACTTCGCG

                                             150       160       170       180       190
1. Crithidia mellificae strain SF GAPDH TACCAGATGAAGTTCGACACGGTGCACGGCCGCCCGAAGTACACGGTG
2. Crithidia mellificae JF423199        UACCAGAUGAAGUUCGACACGGUGCACGGCCGCCCGAAGUACACGGUG
3. Crithidia mellificae AB716357
4. Crithidia mellificae AB745489        TACCAGATGAAGTTCGACACGGTGCACGGCCGCCCGAAGTACACGGTG

                                               200       210       220       230       240
1. Crithidia mellificae strain SF GAPDH GAGGTTGCGAAGAGCTCGCCTGAGGTGAAGAAGCCGGACGTGCTCGTG
2. Crithidia mellificae JF423199        GAGGUUGCGAAGAGCUCGCCUGAGGUGAAGAAGCCGGACGUGCUCGUG
3. Crithidia mellificae AB716357                                       AGCCGGACGUGCUCGUG
4. Crithidia mellificae AB745489        GAGGTTGCGAAGAGCTCGCCTGAGGTGAAGAAGCCGGACGTGCTCGTG

                                                 250       260       270       280
1. Crithidia mellificae strain SF GAPDH GTGAACGGCCACCGCATCCTGTGCGTGAAGGCGCAGCGCAACCCGGCG
2. Crithidia mellificae JF423199        GUGAACGGCCACCGCAUCCUGUGCGUGAAGGCGCAGCGCAACCCGGCG
3. Crithidia mellificae AB716357        GUGAACGGCCACCGCAUCCUGUGCGUGAAGGCGCAGCGCAACCCGGCG
4. Crithidia mellificae AB745489        GTGAACGGCCACCGCATCCTGTGCGTGAAGGCGCAGCGCAACCCGGCG

                                         290       300       310       320       330
1. Crithidia mellificae strain SF GAPDH GACCTGCCGTGGGGCAAGCTCGGCGTGGAGTACGTGATCGAGTCGACC
2. Crithidia mellificae JF423199        GACCUGCCGUGGGGCAAGCUCGGCGUGGAGUACGUGAUCGAGUCGACC
3. Crithidia mellificae AB716357        GACCUGCCGUGGGGCAAGCUCGGCGUGGAGUACGUGAUCGAGUCGACC
4. Crithidia mellificae AB745489        GACCTGCCGTGGGGCAAGCTCGGCGTGGAGTACGTGATCGAGTCGACC

                                           340       350       360       370       380
1. Crithidia mellificae strain SF GAPDH GGCCTGTTCACGAACAAGGCGAAGGCCGAGGGCCACGTGAAGGGTGGC
2. Crithidia mellificae JF423199        GGCCUGUUCACGAACAAGGCGAAGGCCGAGGGCCACGUGAAGGGUGGC
3. Crithidia mellificae AB716357        GGCCUGUUCACGAACAAGGUGAAGGCCGAGGGCCACGUGAAGGGUGGC
4. Crithidia mellificae AB745489        GGCCTGTTCACGAACAAGGTGAAGGCCGAGGGCCACGTGAAGGGTGGC

                                             390       400       410       420       430
1. Crithidia mellificae strain SF GAPDH GCGAAGAAGGTGGTGATCAGCGCGCCGGCGTCTGGCGGCGCGAAGACG
2. Crithidia mellificae JF423199        GCGAAGAAGGUGGUGAUCAGCGCGCCGGCGUCUGGCGGCGCGAAGACG
3. Crithidia mellificae AB716357        GCGAAGAAGGUGGUGAUCAGCGCGCCGGCGUCUGGCGGCGCGAAGACG
4. Crithidia mellificae AB745489        GCGAAGAAGGTGGTGATCAGCGCGCCGGCGTCTGGCGGCGCGAAGACG

                                               440       450       460       470       480
1. Crithidia mellificae strain SF GAPDH ATCGTGATGGGCGTGAACCACCACGAGTACGACCCGGCCACGCACCAT
2. Crithidia mellificae JF423199        AUCGUGAUGGGCGUGAACCACCACGAGUACGACCCGGCCACGCACCAU
3. Crithidia mellificae AB716357        AUCGUGAUGGGCGUGAACCACCACGAGUACGACCCGGCCACGCACCAU
4. Crithidia mellificae AB745489        ATCGTGATGGGCGTGAACCACCACGAGTACGACCCGGCCACGCACCAT

                                                 490       500       510       520
1. Crithidia mellificae strain SF GAPDH GTCGTGTCGAACGCGTCCTGCACGACCAACTGCCTGGCCCCGATCGTG
2. Crithidia mellificae JF423199        GUCGUGUCGAACGCGUCCUGCACGACCAACUGCCUGGCCCCGAUCGUG
3. Crithidia mellificae AB716357        GUCGUGUCGAACGCGUCCUGCACGACCAACUGCCUGGCCCCGAUCGUG
4. Crithidia mellificae AB745489        GTCGTGTCGAACGCGTCCTGCACGACCAACTGCCTGGCCCCGATCGTG

                                         530       540       550       560       570
1. Crithidia mellificae strain SF GAPDH CACGTGCTGACGAAGGAGAACTTCGGCATCGAGACCGGCCTGATGACG
2. Crithidia mellificae JF423199        CACGUGCUGACGAAGGAGAACUUCGGCAUCGAGACCGGCCUGAUGACG
3. Crithidia mellificae AB716357        CACGUGCUGACGAAGGAGAACUUCGGCAUCGAGACCGGCCUGAUGACG
4. Crithidia mellificae AB745489        CACGTGCTGACGAAGGAGAACTTCGGCATCGAGACCGGCCTGATGACG

                                           580       590       600       610       620
1. Crithidia mellificae strain SF GAPDH ACCATTCACTCCTACACGGCGACGCAGAAGACGGTGGACGGCGTGTCG
2. Crithidia mellificae JF423199        ACCAUUCACUCCUACACGGCGACGCAGAAGACGGUGGACGGCGUGUCG
3. Crithidia mellificae AB716357        ACCAUUCACUCCUACACGGCGACGCAGAAGACGGUGGACGGCGUGUCG
4. Crithidia mellificae AB745489        ACCATTCACTCCTACACGGCGACGCAGAAGACGGTGGACGGCGTGTCG

                                             630       640       650       660       670
1. Crithidia mellificae strain SF GAPDH CTCAAGGACTGGCGCGGCGGCCGCGCTGCTGCGGTGAACATCATCCCG
2. Crithidia mellificae JF423199        CUCAAGGACUGGCGCGGCGGCCGCGCUGCUGCGGUGAACAUCAUCCCG
3. Crithidia mellificae AB716357        CUCAAGGACUGGCGCGGCGGCCGCGCUGCUGCGGUGAACAUCAUCCCG
4. Crithidia mellificae AB745489        CTCAAGGACTGGCGCGGCGGCCGCGCTGCTGCGGTGAACATCATCCCG

                                               680       690       700       710       720
1. Crithidia mellificae strain SF GAPDH AGCACGACCGGCGCCGCCAAGGCCGTGGGCATGGTGATCCCGTCGACG
2. Crithidia mellificae JF423199        AGCACGACUGG
3. Crithidia mellificae AB716357        A
4. Crithidia mellificae AB745489        A

                                                 730       740       750       760
1. Crithidia mellificae strain SF GAPDH AAGGGCAAGCTGACCGGCATGTCCTTCCGCGTGCCGACGCCGGACGTG
2. Crithidia mellificae JF423199
3. Crithidia mellificae AB716357
4. Crithidia mellificae AB745489

                                         770       780       790       800       810
1. Crithidia mellificae strain SF GAPDH TCCGTGGTGGACCTGACGTTCCGCGCGACCCGCGACACGTCCATCCAG
2. Crithidia mellificae JF423199
3. Crithidia mellificae AB716357
4. Crithidia mellificae AB745489

                                           820       830       840       850       860
1. Crithidia mellificae strain SF GAPDH GAGATCGACGCTGCCCTGAAGAAGGCAGCCAAGACGTACATGAAGGGC
2. Crithidia mellificae JF423199
3. Crithidia mellificae AB716357
4. Crithidia mellificae AB745489

                                             870       880       890       900       910
1. Crithidia mellificae strain SF GAPDH ATCCTGGGCTACACGGACGACGAGCTTGTGAGCAGCGACTTCATCAAC
2. Crithidia mellificae JF423199
3. Crithidia mellificae AB716357
4. Crithidia mellificae AB745489

                                               920       930       940       950       960
1. Crithidia mellificae strain SF GAPDH GACGCCCGCAGCTCCATCTACGACTCGAAGGCAACGCTGCAGAACAAC
2. Crithidia mellificae JF423199
3. Crithidia mellificae AB716357
4. Crithidia mellificae AB745489

                                                 970       980       990       1,000
1. Crithidia mellificae strain SF GAPDH CTGCCGGGCGAGAAGCGCTTCTTCAAGGTTGTGTCGTGGTACGACAAC
2. Crithidia mellificae JF423199
3. Crithidia mellificae AB716357
4. Crithidia mellificae AB745489

                                         1,010    1,019
1. Crithidia mellificae strain SF GAPDH GAGTGGGGCTA
2. Crithidia mellificae JF423199
3. Crithidia mellificae AB716357
4. Crithidia mellificae AB745489
```
