# Supplementary material for: A Draft Genome of the Honey Bee Trypanosomatid Parasite Crithidia mellificae
Source: PLoS One. 2014 Apr 17;9(4):e95057. doi: 10.1371/journal.pone.0095057 (PMC3990616; doi:10.1371/journal.pone.0095057)

Supporting Figure S2. Nucleotide alignment (799 nt) of the glyceraldehyde 3-phosphate dehydrogenase (GAPDH) gene. The alignment was performed on the Geneious software workbench, using the ClustalW aligner.

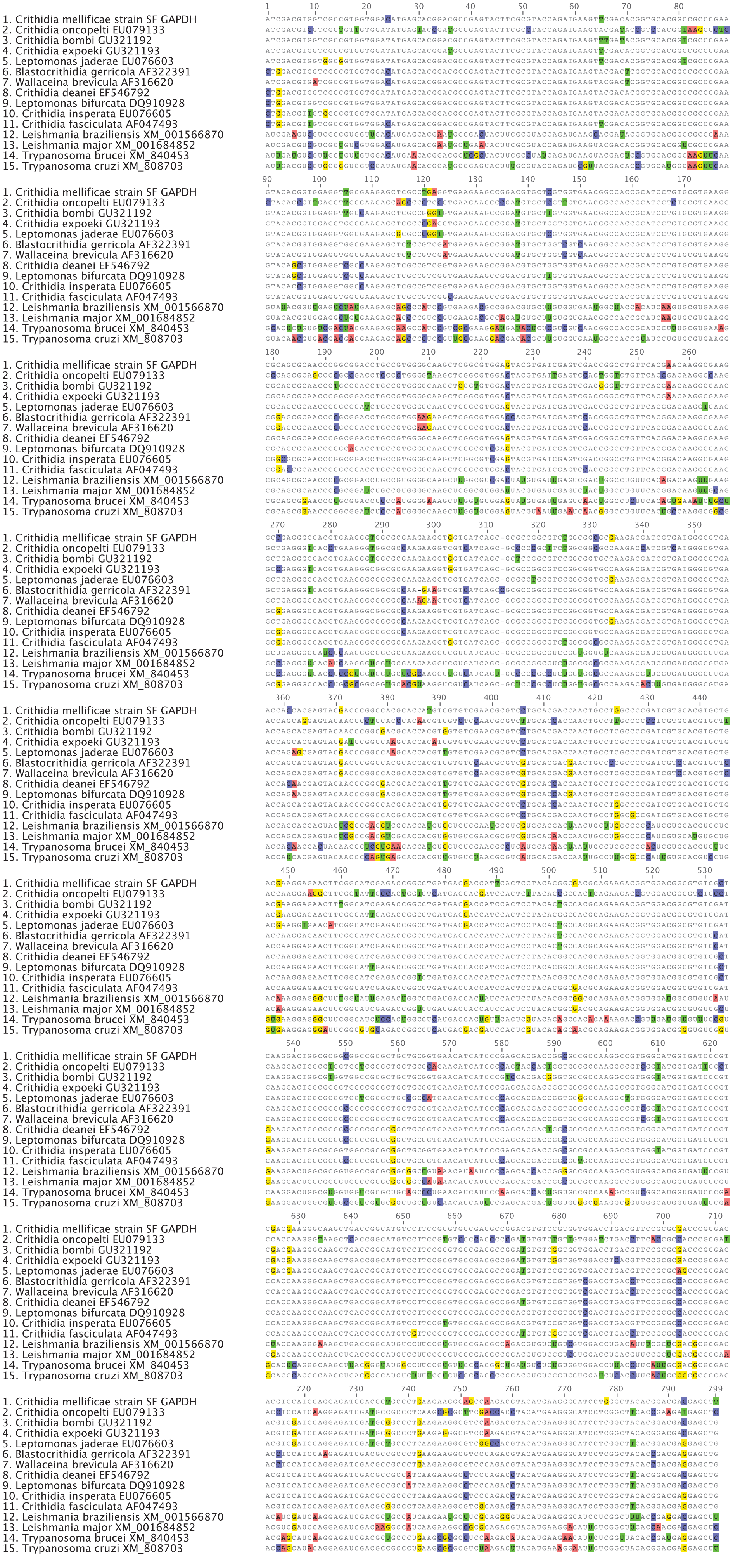

Supplement: Figure S2 — Nucleotide alignment (799 nt) of the glyceraldehyde 3-phosphate dehydrogenase (GAPDH) gene. The alignment was performed on the Geneious software workbench, using the ClustalW aligner. (PDF) [file pone.0095057.s002.pdf]
