# Supplementary figures and images for: A Draft Genome of the Honey Bee Trypanosomatid Parasite Crithidia mellificae
Source: PLoS One. 2014 Apr 17;9(4):e95057. doi: 10.1371/journal.pone.0095057 (PMC3990616; doi:10.1371/journal.pone.0095057)

Supporting Figure S3. N-value assembly metric of the *C. mellifica* draft genome.

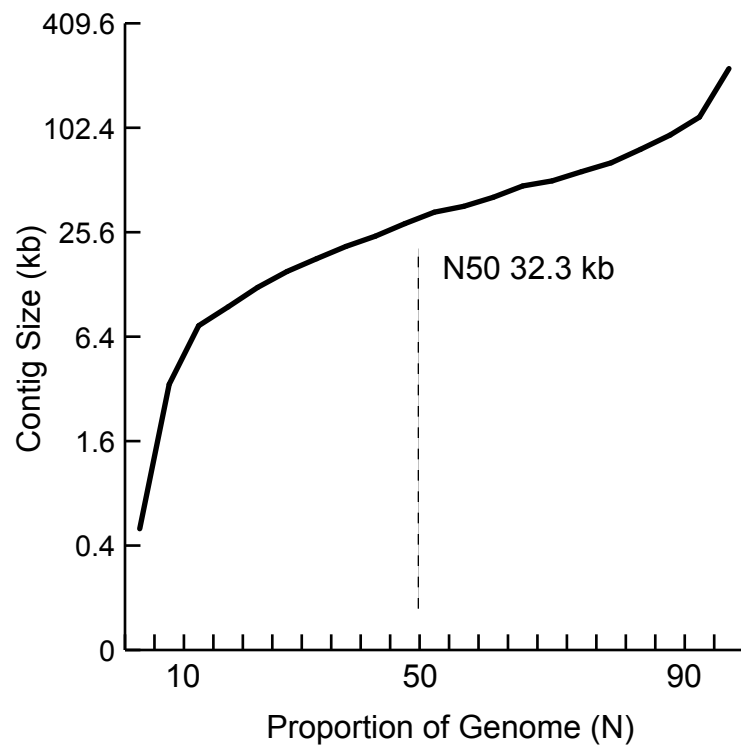

Supplement: Figure S3 — N-value assembly metric of the C. mellificae draft genome. (PDF) [file pone.0095057.s003.pdf]
