# Supplementary material for: A Draft Genome of the Honey Bee Trypanosomatid Parasite Crithidia mellificae
Source: PLoS One. 2014 Apr 17;9(4):e95057. doi: 10.1371/journal.pone.0095057 (PMC3990616; doi:10.1371/journal.pone.0095057)
Supplement: Figure S4 — C. mellificae orthologs of spliced L. major genes. (PDF) [file pone.0095057.s004.pdf]

Supporting Figure S4. Crithidia mellificae orthologs of spliced L. major genes

LmjF.29.2600 poly(A) polymerase, putative
>CrithidiaMellificaeGenomeContig_672_-_CDS CrithidiaMellificaeGenomeContig_672
TTGTGGGTTGTGCTGCACGTTCCATATGCATACGCGTCCTTCGTCACACACGCACACAAACGTGCACAAAACGAATGTACTTGGTTGTATAGAATTGCGTTGTGTTCGGCA
ACGATTGATGTTAATCTCTTTTTCACCAAGCTTCCCCAGTTTCTCAACGGGCGTCTTTCCGACCTCGTCGTCGTCACTTCCACCCGCGTCCCGCTCCTCAAGTTCAGCTAC
CACACGGTGCACGTCGACCTCCTCTTCGCTTCTGTCGATCTTGCCGAGCCGCCGAGCAGCGCCGATCTTCTCCAGGACAGCTTTCTCTTACGCGTGTCGGAGAGCAGTCGT
GCCACCGTCAATGGCATCCGCACCATCCTGGAAATTCACCGAAGACTGCGCCTGCCGCAAGACGACTACGCCTGCGTCCTTCGCGCCGTCAAGTACTGGGCGGCGCGGCGA
CAAGTCTACGGCAACCTGTACACCTTTCCCAATGGCGTGTGCCTCGCCATAATGGTGGCTCGTGCCACGCAGCTGTGCCCGGTGCAGGACCCGAGCGCGTTGCTGCGGTTC
TTTTTTGCGTTCTACGTGCGGTGGCTCTCGCGTAGGTCCCGCATCGCGCCGATCACGATCGTTTCGGCCGAACACGCGACGACCATGGCTGCCGTGCCTGGCATGCCGCGT
GCGTGGGACGCCGTGTGTGACGCGACAGACCTTCTCCCGATTCTCAACCCGGCCCGTCCTTCGGTGAACGCCGCCTATACCGTCGGCCGCAGCGGGCTGCAGCTTTTTTAC
CAGGAACTGCGACGCGCCGATGAGCTGCTGCGTGGGGCATCGGCCGACACGCCACCGTACCGCGAACTGTGGCGGCCGTACGATCTCCTGCAGGAGTACCGCTACTTCATC
GGCGTCCACATCGCCAGCGTGCACGCGTCGCCGACGGCGTGCGAGAACGTGCTAAACGCGTGGAAAGGCTACGTGGAGAGCAAGCTACGCATGCTTATCTACGCTCTCGAG
TGTGTCGCGGAGGTGCGGCCCTTCCCACAGTGCGTGGTGGACACCTCTAGCCAAGAAATCAACGAGGATGGCGCTATTCTGCGACAGTCGTCACGTGTGTACTTCTTCGGT
GTGCGGAGCGGCGATGCAACGCTGCATCGAGACATGTTTGCGAGCGCGTTTCGTGAGTTTGAGTACGCGGTGGAGGAGGGCACTTCGCCACGGTTCGGCTTCGTGCGTGAC
GTGGACAGCATGCGTGGACCGTGGTTCTCATTTTCGGTCAAGGAGGAGGACGAGGAGGGAATGACGACATCTCCGCCGAGGTCTGCGTTGCGCACACTGCAGTCTGCCTGT
GCGAGGTTCACGCTAGCAAGCGTGTGA

LmjF07.3400 ATP-dependent DEAD/H RNA helicase
>CrithidiaMellificaeGenomeContig_35_-_CDS CrithidiaMellificaeGenomeContig_35
ATGTCCGACTACGGCGGCGGGTACCGACGTGGTGGGGGCGGCGATGGCGGCGGCTATGGCGGCGGGCGCGGACGTGGTGGCTACGGTGGCTACGGCCGCGGTGGTGGCTAC
GGCGGGGGCTACGGCCACGATCACCACAGCAACGGCGGTCTTGGCGCGAATCTACACCGTATAGACTGGGAGGCGGTGCAGAAGGTGGCGACGCAGTGGAACTTCTACAAG
CCGCAGAAGCCGCGCAGCGAGGAGGAGATCGCGCAGTGGCTGCGCGAGAACAGCATCACCATCTACGGCGATCGCGTGCCGCAACCGATGCTGGAGTTTTCGGACCTTGTC
GCGCCCGACGCGATTCACCAGGGCTTCGCGGATGCAGGGTTTGTGAAGCCGACGGCTATCCAGTCCGTCGCCTGGCCGATCCTGCTCAACTCGCGCGACATCGTCGGTGTG
GCCAAGACGGGCTCTGGCAAGACCATGGCCTTCATGATCCCCGCCGCGTTGCACATCATGGCACAGCCGCCGCTGCAGCCCGGCGACGGCCCCATCGCCCTCGTCCTCGCC
CCCACGCGCGAGCTGGCGGTGCAGATCGAGACGGAGACCCGCAAGGCGCTTACCCGGGTGCCGAACATCCTGACGACATGCGTGTACGGCGGTACGCCGAAAGGCCCGCAG
CAGCGCGCGCTGCGTGCCGGCGTGCACGTCGCGATTGCCACGCCTGGCCGTCTGATCGACCTGCTGGAGACAAACTGCACCAATTTGCTGCGCATCACCTATCTGGTCCTC
GACGAGGCGGACCGGATGCTGGATATGGGCTTCGAGGACCAGATTCGCAAGATATGCTCGCAGATCCGCACGGACCGGCAGACACTCATGTTCTCCGCGACATGGCCGCGC
GAGATTCGCAATTTGGCGGCGAGTTTCCAGAAGGACTTCGTCCGCGTGCACATCGGTTCCGAGGAGCTCGTGGCGAACGCTGACGTTCACCAGCACGTCTTGGTGGTGGAG
GGCTACCACAAGGAGGAAAAGTTGGAGGAGATTCTGCGCCAAGTAGGACCGCAGCGTGTGCTGATTTTTGTCAAGACGAAGAAGTCCGCGGATATCTTGCAAGACCGTTTG
GGCCGTGCGCTGCGTCAGACCGTCTTGGCGATCCACGGCGACAAGCTGCAATCAAGCCGCGACTACGTCCTCGACCGCTTCCGCAAGGACAGCCGCGCCATTCTCGTTGCC
ACCGATGTGGCGGCCCGTGGGCTTGATATCAAGGACCTGGACGTGGTCGTGAATTACGATATGCCACTCAATATTGAAGATTACGTGCACCGTATTGGTATGTTGAACGGG
TTTCCATCGAGCCTACTCCCTTTCTCCCGGTCTTCGATAGCTCCTCTTCCTTTTTTTTTTTCTCTCACTTCTTCTTTTTGGTTTGCTCGTGCCTGCATTGCTTCCGCTACT
TCTTTCATTCAACCTTTTCATCATGCGCTTTTTCTGCGTGTCGCCTTCTCCTAG

LmjF29.2000 hypothetical protein, conserved
>CrithidiaMellificaeGenomeContig_47_-_CDS CrithidiaMellificaeGenomeContig_47
ATGAAGGCCTTGCTCACCTCCGCAGAAACGGCAACACCGTCTGCGCCGCCGTGTTCTGTCGAGTACCCGGCGACCTTCACTGCTGAGCTGATCGAGCACTGTCGGCAGGGC
TGCATCCCGCCGGAGGTCTTCGCGGACGCGCGTGCGCTCCTGGCGGCGGAGCAGCAGCGGCGGCGTTACGTCCGCGTGAATCCGCGATGTCTGCAACGCGTGATCGACGCT
GGTGTGGAAAACGTGCGGCGTCTGGACAGCCTCTCCTCTGCGTCAGCTACGGCGTCGTCATTGATAGATGAAGAGCGGCAGCAGTGGCATGAGCTGCAGCCCTGTCTTTAC
TCTGTTCCGTCCACAGGAGCAAGTGGCGAAGAAGAAAATCGCACGTCAAGTCCTCCCTCCCCGAGCTCCCTCGTACGACTCGGCACAGAGCAGATCGGCGCGCGCGTTCCC
CAGTCCGTTCTTCTTCGGCTTGGCGTTGCGAGGCTCACCGGACTGCCGCTGGCCTCGGTGCAGCCTGTCTTGTGGTTGCCCTGCGGTGTTTTTGCGCTCCCGTGGTCCTAC
GCAATGGGCAGCAGCCCTCTGTTTCGCGAGGGTCTTCTTCTCGCGATGGATGCGGCGTCGGTGGCGGCGGTGGCGGCGCTGCGACCGCGACCCGGAGAGCGGGTGTTGGAC
CTGTGCTGTGCGCCTGGCATGAAGCTGGGCCTCATAGCCGACGCAGTCGCCCGCCCCGACCGCGATCGCCACCCGGTAGACGGAGCAACTCTTCTTTCGCAAGAAGGTCTA
GCAGTGGGCGTGGATGTGAGTCTGCCACGTCTGTACACGACGCGTGCCATGCTCAAGAAGCAGCAGGGCAAGTGTGGCGGCTATGCTGCCTCACCGCCGCCGTCCTTGCCT
GTTTGCCTCTTCAGGGGCGACGGACGCTGCGCCACGATGGCGGACGCGGTAGCGTCGATGAATGACAAAGCAATGGCGGACGTCAGCACCGGCCTCACCACACGAGAGCGG
CGCACGTTGCAGCGGCTGCCAGAGCACGGCGGCTCCAAGCGGCCTCGTCGGGAGTCTGTCAAGGCAGCAGCGGAAAACACATCAACGTCGAGTGAGGCGTCCGACAGCATG
GCAGGTATGTACGTTGTCTACGCTCCTTCAGAGGCACGCGCTTCAATCGCAGCGTGGCAGCAGCAGCAACAACAACAGCAGCAGAGACACGAATGTGAAACCTGTCCTGCT
TCAGCAGCATGGCTCTTTGACCGTGTTTTGGTGGATGCCGAGTGCTCACACGACGGCTCCGTTTCACACATCCAGCTGGAGGACGGCGAGGCACCGGTCGAGACTTCGCAA
GTCGCCTCCGTCGTCAGAAAAGGGAGAGGGCTCACGAACGAGCACCGTATGCAGCACCTCAACTTAGGGACAGGGCCTTCTGCACAATCAGCCTCAGCCGACAACACGCCC
TCCGCTCGTGCGGCGACTTTTTCCTCCTCCTCTCTGTTTGATGTTCAGTCGGCGTTGCTCTACAACGGCTACCAGCAGCTAAAACCGGGTGGTACCCTTGTCTACGCCACC
TGCTCCTACGCGTTTACACAAGATGAACTCGTTGTGCGCCGTTTCCTAGATCGCGTGAATACACCGGAGGAAAAAGAGAAGACTGCCACGCAGACGACGGCAGTTCTCGTG
CCTGCGTTTTCGTTTTCTCACGAGGAAGAACCACAAGACTGTGGTGGTGGTGGTAGTGAGGGCACGCAAAAGCACACGATCTCATCATGCGTCTGCATGGAGAGCGCTGCC
GCTGAGGAGGGCCTGCAGCAACTCCTTGACGCGCACGCCGCAGACTACGGCGTGGTGGAAAGTGGGATGCGTCGCTACTTCCCACAGGAGACAGCGTCGCCATCTGTGGCA
GTTGGCAGTCGCTTCTGGCCGCGGACGTTTTCTAGTAGCTTCCTTTACGTGGCGAAGATATGGAAGAAGGTGGGGTAA

LmjF32_0850 RNA-binding protein, putative
>CrithidiaMellificaeGenomeContig_774_-_CDS CrithidiaMellificaeGenomeContig_774
ATGGCGGAGCGCAATTACAGCCCCTTCAGCGGCTTCTCTAGCGGCCACGCAAACGGCAAAGAAAAAGTTGGGGCGATGGGTAGCAAGCTCATGCCGGTCGACTGGTCCGCC
AAGACGCTCGTCCCGGGCAAATGGAAGGTGGTCGACGCCAACGCCATCCGCAAGGCTGCCAGCGTGAAAGACGATCACGGCGCCAGCAAGGTGAAGCAGCTCTCCGCCGCG
GAGGCGGACGAGTGGCGCCAGACGCATTCCATCACCATCTCCGACGCCGACGACTGCCCGGCGCCGTTCACGCAGTTCGGGATGCTCACCTCCGTCCCTGCCTACCTCAAG
GCGAAGCTGCTGGCCCAGGGCTTCCAGGCCCCCACACCGATCCAGGCGCAGTCGTGGCCGATCGTGCTGTTGGGCCGCGACCTTGTCGGCGTGGCTAAGACCGGCTCTGGC
AAGACCCTTGCCTTCATCATCCCGGCACTGGCCCACATCGCACTGCAGGAGCCGCTGAAGGTGGGTGACGGTCCCATGGTGATTGTCCTCGCTCCCACGCGCGAGCTGGCG
CAGCAGATCGAGCAGGAAACGGTGAAGGTGCTCCCGCAGAGTGTCCGCTGTGGCTGCATCTACGGCGGAGCGCCAAAGGGCCCACAGCTGGGAATGCTACGCCAGGGTGTC
CACATCCTCGTCGCCACACCGGGCCGTCTCATCGACTTTATGGAAATCAAGCGCGTAAATCTGCTCCGTGTGACCTACCTCGTACTGGATGAGGCGGACCGAATGCTGGAC
ATGGGCTTTGAGCCGCAGGTGCGCGCCATCTGCGGACAAATCCGCCCGGACCGCCAAACACTCATGTTCTCCGCCACCTGGCCGCGTGAGATTCAGAACCTGGCGGCCAGC

TTTCAGAAGAACTGGATCCGCATCAACGTCGGCAGCATGGAGCTGCTGGCAAACAAGGATGTCACGCAGAACTTCATCCTGACGACCGAGGCGGGCAAGCTGGACGCGTTA
CACAAGCTGATGGAGCGCCACCGCAACCAGCGCGTGCTGGTCTTCTGCAAGACAAAGAAGACAGCCGACTACCTCGAGTACCAGCTGAAGCGTAGCGGCGTTGACGCGATG
GCGATCCACGGCGACAAGGAGCAGCGCCAGCGTGAGTTCATCCTCGAGCGGTTTCGCAAAGACCCCCGGCTGTGCGTCGTGGCGACGGACGTGGCAGCGCGTGGGCTGGAC
ATCAAGGATTTGGAGACGGTCGTCAACTATGACTTCCCCATGCAGATCGACGACTACGTGCACCGCATTGGTCGCACCGGTCGCGCAGGCGCAAAAGGCGCGGCCTTCACG
ATGATCACGAAGCAGGAGAGTCAGCTGAACCCGCCCACCGTGCGGCAGCTGGTGGGACTGGTGGAGCGTGCCGGACAGGAGGTGCCGTCATGGATGCGGGAGTGGGCGGAC
CAGGGCAGCGGCTTCGTCGCGATGAAGCGCAACCGCAACATGATGGGCAGTTTTGACCGCAATGGACCGCGAATGCGCATGCCGGGCGACCGTCCCACCCACTCGGCGAGC
GGCAGCGCGGGCCATTTTGGCCTAAAACCGCATGGCAGCGGAGCGCAGGCCTTCGGCATGAACGGCGGCGGTGCGACAGGCATTCAGAATAAGAAGTTCGACTATAGCAGC
GACGAGGACGAGCATCCGGCAAAGCGCGCTCGTGAGTAG

LmjF32.0850 conserved hypothetical protein
>CrithidiaMellificaeGenomeContig_1343_-_CDS CrithidiaMellificaeGenomeContig_1343
ATGTCTACCATCGTGCTGCACAACCTCCCGGTAGGGTGCATGGAGTCCGATATTCACCGCGCGCTGCTGATTTACGGCACGGCGGTGAATATAGAGATGGACACGCGCACC
TCGCAGGCGGTGGTCACCATGAGGACAACGCAGGAGGCGCAGGCTCTGCTGAACCGCCACTCCGTGAACGTGAGTGGGACGTCGGTGCGGGTGGACACGCAGGCGTCACAG
CCTCCGCCGCCTGCACCTGCGCCGTCGTTTCCACCGCCCCACATGGCCGCGAACGCATACCCCTTTTCGAGGGTACCGGGCACAATGTACGCTACCGGCATGCCTGCGACT
GTGTCTGTGCCGGCGGCGCCTCCGTCGCATTACGCACCATCTTCGCTTCTGCCTTCTAGCAGCAGTGGCGGCGGCGGTGGTGCCATCATGCCAGCTCCACCGCAGACGTCG
CGGCGGCTGAAGGTGGTGGTCGAGGATTGCCGCTACCCCATCACACGCGACGTGCTGCTGCGCATGTTCAGCATGATCGCCCCTCCCGTGATGGTCACCTGCGGTCCGTAC
GGACCCACGACGACGGGGATGGTCGAGTTTGCCGAGGCCGGCGCGGCGCAGCAAGCAGTGGACCAGTTCCACAGCAAAGCCATCTACCCGGATTGCTGCTACGTGAAGCTG
TCCTTCGAGCCCATGTGGGAAGTCCCGGTGAGCACGACGCCACCAGCCGTGGGCGGCGGTGCAGGGGGCCCCACTCGTACCGCGTACCCGTACGCCGCGCAGCCGAGCATC
CCGGGCAATACAAGCCCCTACGCCGCGCCACCGAGCACAGACGCCACCGCGCGCTACCCGGGCTGGGACGGCCGCGATGACGCGCCGTACTACCGAGGCGGCGGCAGCCGC
GAGGGCTCCAATATCCCCTTCCCTCCTGCCATGGGCGGGCGGGAGCAGGCCCACGGCGACGACGGACGGCGGGAGTACGTTGTCGCCGGTGGCCGAGGCGGCGGTGGCCCA
CACGGGCTGATGATACGCGGTGGGCGTGGCGCCGCGCGTGGTGCGATGCGAGGCGGGGGCATGCCTGGCACAGCTGTGGGCTTTAGCCCGTACGATACCACGCCGTTCCCT
CCCCACCATCCCGCCTCGTCGCGGGCAGCAGGGGAGCACGCGGGGCCCGGCGTTGGGAGCGGCCGCCCCGCTGCCCCGCCTGCGACCGACTGCGCCGTCCTCGTCTCTGGC
GTGGCGGAATCAGTTCCGCTCTACGACTTATGGGTGCTGCTGGAGGTGTATGGCAACGTGAAGTCGCTCAAGCGGCAGCACACGGACCACAGGCAGGTCGTGGCACAGTTT
CAACACGCAACGGACACGGTGCTCGCGGTGCAGTACCTGCACGGCTGCCCTTTTCGCGGGATGAAGCTGCGTCTGAAGCGCTTCCTCGGCTACCATGAGCGCAACACGGAG
TGGAACTTGGGCCCCGCCACGGACCCGTCCACGTTGGCGGCGCTCTTCGAGACCGGCTACCACCACCGCGTTGCCCCACACGTGCCACGCAACCCGCACAGCGCGATGTAT
CCTGACAAGAACGTCTTCGTCAGCAACCTGACGGAGGCGATCACGGACTATGAACTCGAGGAGATGTGGCGGCGGCTCGGCTTTGAGCCGATCGCGTCGTACCGCCGTGGG
CCGAAGGCGTCCATCGTCGGCTTCAAGGACATCGAGACGGCCGTGAACGCACTCATTGCGGTGCATCTATCTGACTGTCATGGCCGCACGCTCTACGCCACCTTTTCGCGC
TTCCCACCCGGCCCGCGGCCCCCCAAGTCGGCGGACGACGATGCGCCGGACGGGGAACAGAATGACGACGACCCGCAGCAGGAACAGGGGGAAGAGGCAGGTCAGGCAAAG
CAGGATGCGGTAAAGCCGCCAGCGACCGCCGAGGATGCCTCGGCGAACGTGTCTGCGCCAGCGGAGAAGGCAGATTCTGTCACGGCTGCCACAGACGAGGCAAAGCCGCCC
TCGAAGGCCGGCAAGCACAAGACGAAAGCTGCAAAGAAGTAG
